# Molecular Mechanism of Equine Endometrosis: The NF-κB-Dependent Pathway Underlies the Ovarian Steroid Receptors’ Dysfunction

**DOI:** 10.3390/ijms23137360

**Published:** 2022-07-01

**Authors:** Tomasz Jasiński, Łukasz Zdrojkowski, Graça Ferreira-Dias, Ewa Kautz, Edyta Juszczuk-Kubiak, Małgorzata Domino

**Affiliations:** 1 Department of Large Animal Diseases and Clinic, Institute of Veterinary Medicine, Warsaw University of Life Sciences, 02-787 Warsaw, Poland; tomasz_jasinski@sggw.edu.pl (T.J.); ewa_kautz@sggw.edu.pl (E.K.); 2 Departmento de Morfologia e Função, CIISA—Centro de Investigação Interdisciplinar em Sanidade Animal, Faculdade de Medicina Veterinária, Universidade de Lisboa, 1300-477 Lisbon, Portugal; gmlfdias@fmv.ulisboa.pt; 3 Laboratory of Biotechnology and Molecular Engineering, Department of Microbiology, Prof. Wacław Dąbrowski Institute of Agricultural and Food Biotechnology—State Research Institute, 02-532 Warsaw, Poland; edyta.juszczuk-kubiak@ibprs.pl

**Keywords:** NF-κB, endometrosis, receptor, estrogen, progesterone, endometrium, horse

## Abstract

Endometrosis is a frequently occurring disease decreasing mares’ fertility. Thus, it is an important disease of the endometrium associated with epithelial and stromal cell alterations, endometrium gland degeneration and periglandular fibrosis. Multiple degenerative changes are found in uterine mucosa, the endometrium. However, their pathogenesis is not well known. It is thought that nuclear factor-κB (NF-κB), a cell metabolism regulator, and its activation pathways take part in it. The transcription of the profibrotic pathway genes of the NF-κB in fibrotic endometria differed between the follicular (FLP) and mid-luteal (MLP) phases of the estrous cycle, as well as with fibrosis progression. This study aimed to investigate the transcription of genes of estrogen (*ESR1*, *ESR2*) and progesterone receptors (*PGR*) in equine endometria to find relationships between the endocrine environment, NF-κB-pathway, and fibrosis. Endometrial samples (*n* = 100), collected in FLP or MLP, were classified histologically, and examined using quantitative PCR. The phase of the cycle was determined through the evaluation of ovarian structures and hormone levels (estradiol, progesterone) in serum. The transcription of *ESR1*, *ESR2*, and *PGR* decreased with the severity of endometrial fibrosis and degeneration of the endometrium. Moreover, differences in the transcription of *ESR1*, *ESR2*, and *PGR* were noted between FLP and MLP in the specific categories and histopathological type of equine endometrosis. In FLP and MLP, specific moderate and strong correlations between *ESR1*, *ESR2*, *PGR* and genes of the NF-κB pathway were evidenced. The transcription of endometrial steroid receptors can be subjected to dysregulation with the degree of equine endometrosis, especially in both destructive types of endometrosis, and mediated by the canonical NF-κB pathway depending on the estrous cycle phase.

## 1. Introduction

Previous studies on the equine endometrium have focused on the molecular mechanisms involved in physiological aspects [1,2,3,4] and the pathogenesis of endometrial diseases, often associated with mares’ subfertility [5,6,7,8,9]. Despite the relentless efforts and new scientific reports, the pathogenesis of nonsuppurative endometritis and endometrosis remains unknown, and routinely available treatment is still undeveloped [3,10,11,12]. Endometrosis, also referred to as degenerative endometrial fibrosis, is the most important clinically silent endometrial disease associated with infertility in mares [13,14]. The fertility prognosis of this condition has been based on the histopathological categorization proposed by Kenney and Doig [15], together with the amendments of Hoffmann and collaborators [13]. Thus, equine endometrial samples can be classified in two ways: firstly, as Kenney’s and Doig’s categories from normal endometrium (I) to mild (IIa), moderate (IIb), and severe (III) equine endometrosis [15]. Another classification proposed by Hoffmann et al. for histopathological types of endometrosis includes inactive-nondestructive, inactive-destructive, active-nondestructive, and active-destructive endometrosis [13]. In the endometria of mares affected by endometrosis, both epithelial and stromal cell alterations occur and become the cause of the degeneration, dilatation, and atypical differentiation of the affected glands [14]. At the beginning of fibrogenesis, periglandular stromal cells synthesize collagen fibers, while with the progression of the fibrotic process there is a predominance of myofibroblasts and metabolic active or inactive stromal cells without signs of collagen synthesis [5]. The epithelial differentiation may present an irregular pattern in fibrotic uterine glands [16], causing alterations in several epithelial cell enzyme patterns [17], and glycoconjugates [18]. As epithelial cell differentiation usually depends on the ovarian steroids’ concentration, the independence of hormonal control and altered paracrine interaction of endometrium is suspected in the pathogenesis of endometrosis [5].

The healthy endometrium undergoes cyclic changes with typical cellular patterns in response to the ovarian steroid hormones estradiol and progesterone [13]. The ovarian steroids act on the endometrium through two pathways, canonical and noncanonical. The canonical mechanism mediates their effects through nuclear receptors, whereas the non- canonical mechanism acts at the membrane level. In the noncanonical pathway, recognized as a nongenomic signaling, steroid hormones nonspecifically bind to extracellular or membrane proteins which may cause a variety of biological effects. In the canonical pathway, recognized as a genomic signaling, steroid hormones specifically bind to intranuclear receptors, which causes a specific biological effect, which is considered to be responsible for functional cyclic changes in the endometrium [19].

In the canonical pathway, estrogen has two specific estrogen receptors (ER-α and ER-ß), encoded by different genes, *ESR1* (locus NM 001081772) and *ESR2* (locus XM 001915519) [20,21]. In turn, progesterone has two isoforms of progesterone receptors (PR isoform A and PR isoform B), encoded by the same gene *PGR* (locus XM 001498494) [21,22]. Both estrogen and progesterone pass through the cytoplasm and bind to an intranuclear receptor [23] in the luminal and glandular epithelial cells and stromal cells [24], and act through the induction of transcription for a variety of genes, thus regulating cell development and differentiation [25]. The binding of estradiol to ER-α, the predominant ER in the uterus [26], stimulates the cellular proliferation of both the epithelial and stromal cells and upregulates PR expression [3,21,27]. On the contrary, the binding of estradiol to ER-ß inhibits the uterotrophic effects of ER-α and downregulates the PR expression in the luminal epithelium [26]. Similarly, progesterone binds to PR, with isoform A being predominant in the uterus and ovaries, while PGR-B is mostly expressed in the mammary gland [28], antagonizes estrogen-induced epithelial proliferation and downregulates PR expression [3,27]. In this way the circulating concentrations of ovarian steroids regulate not only biological effects, but also the abundance of their receptors [21,23,24,29]. In estrus, a higher endometrial expression of ER-α, but not ER-ß, was evidenced compared to diestrus [21,29]. However, the expression of PR differed similarly to ER-α [29] or did not differ [21] between the phase of the estrous cycle. Moreover, the abundance of both ER and PR varies with the type of endometrial cell [21,23,24,29]. In glandular epithelia, the highest levels of ER and PR were observed in the early diestrus, whereas the lowest was found in the mid-diestrus. In contrast, in stromal cells, receptor levels gradually increased from the lowest in the early diestrus to the highest on the ovulation day [24]. Thus, stromal proliferation is most strongly expressed in proestrus, and epithelial proliferation is most strongly expressed in early diestrus [21,24,29]. Both differences in the abundance of ER and PR, which are estrous phase- and cell type-dependent [21,23], cause hormone-induced morphological and functional changes in the endometrium. These hormone-induced changes are mediated by paracrine factors and seem to be dependent on the crosstalk between epithelial and stromal cells [3,30].

In mares’ endometria affected by endometrosis, the damage of the basal lamina and a disturbed stroma–epithelium interaction [14] may cause the deregulation of the effect of ovarian steroids on the glandular epithelium, as it is mediated by the surrounding stroma [13,14]. Moreover, endometrotic glands often display an abnormal abundance of both ER and PR. In contrast to healthy glands, predominantly low ER and PR expression in affected endometrium was evidenced [5,13,14,17,24]. This fibrosis-associated abnormal abundance of ER and PR, as well as alterations in the cytoskeleton and basement membrane [13,14,16], seems to be involved in changes in the components of histotrophe in endometrotic glands, thus impairing fertility [3,5,14]. Besides the ovarian steroid receptors, the abnormal expression of cellular differentiation markers (desmin, vimentin, α-smooth-muscle-actin, and Ki-67-antigen) [1,13], basement membrane integrity markers (laminin, and fibronectin) [13], endometrial proteins (uterocalin, uteroferrin, uteroglobin, and calbindin) [5], and subunits of the nuclear factor kappaB (NF-κB) pathway (RelA, RelB, NF-κB1, and NF-κB2) [31,32] have previously been evidenced in the endometrotic endometria. The NF-κB activation takes place through canonical and noncanonical signaling pathways, which engage RelA/NF-κB1 and RelB/NF-κB2 subunits, respectively [33]. Each activation of this pleiotropic transcriptional regulator affects the transcription of genes of many proteins involved in fibrotic, inflammatory, and defense processes [31,34,35,36] in many fibrosis-related diseases [37,38,39,40,41]. In equine endometrium, the transcription level of the investigated NF-κB subunits and the NF-κB-pathway-related pro-inflammatory molecules (MCP-1, monocyte chemoattractant protein-1; IL-6, interleukin-6) and hyaluronan synthases 1–3 (HAS1; HAS2; HAS3) differed significantly not only in terms of the degree of Kenney and Doig’s [31] and Hoffmann et al. [32] endometrosis classification, but also in terms od the phase of the estrous cycle [31,32]. Therefore, we hypothesized that endocrine-related morphological and functional endometrial disturbances, which were suspected to be one of the mechanisms of mares’ infertility [3,13,24], could be related to the NF-κB signaling pathway.

The present study aimed at the comparison of estrogen and progesterone receptors’ transcription levels in equine endometrium between Kenney and Doig’s categories [15] and histopathological types [13] of equine endometrosis, considering the expression of selected genes (*RelA*, *NK-κB1*, *NK-κB2*, *MCP-1*, *IL-6*, *HAS1*, *HAS2*, and *HAS3*) involved in the NF-κB signaling pathway.

## 2. Results

### 2.1. The Categories and Histological Types of Equine Endometrosis and the Ovarian Steroid Receptors

**Figure 1:**
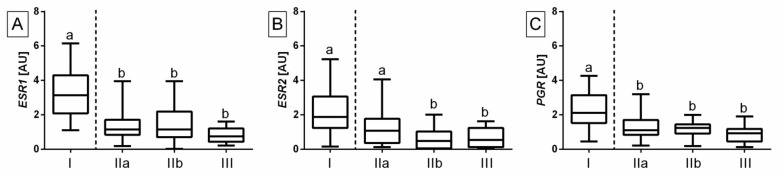
Transcription levels of (**A**) estrogen receptor α (*ESR1*), (**B**) estrogen receptor ß (*ESR2*), and (**C**) progesterone receptor (*PGR*) in the mares’ endometria. The endometrial samples were classified as Kenney and Doig categories I (*n* = 20), IIa (*n* = 30), IIb (*n* = 30), or III (*n* = 20). Category I included C group (*n* = 20) samples; category IIa included types E IN (*n* = 5), E ID (*n* = 10), E AN (*n* = 12), and E AD (*n* = 3) samples; category IIb included types E IN (*n* = 11), E ID (*n* = 5), E AN (*n* = 5), and E AD (*n* = 9) samples; and category III included types E IN (*n* = 4), E ID (*n* = 5), E AN (*n* = 3), and E AD (*n* = 8) samples. Boxes represent lower quartile, median, and upper quartile, whereas whiskers represent minimum and maximum values. The dashed line separates the categories of healthy from affected endometrium. Lowercase letters indicate differences between categories of endometrosis for *p* < 0.05.

The transcription of the studied genes regarding categories of equine endometrosis was higher in category I than in categories IIa, IIb, and III for *ESR1* (*p* < 0.0001; Figure 1A), *ESR2* (*p* < 0.0001; Figure 1B), and *PGR* (*p* < 0.0001; Figure 1C), except for *ESR2* transcription in category IIa, which was similar to category I. No differences were found between categories in the affected endometrium for the three studied receptors, excepting difference in *ESR2* transcription between category IIa and categories IIb and III (*p* < 0.05).

**Figure 2:**
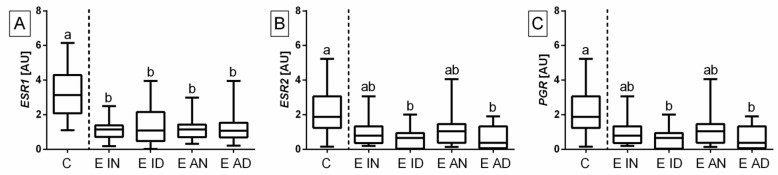
Transcription levels of (**A**) estrogen receptor α (*ESR1*), (**B**) estrogen receptor ß (*ESR2*), and (**C**) progesterone receptor (*PGR*) in the mares’ endometria. The endometrial samples classified as control group (C; *n* = 20) or as inactive nondestructive (E IN; *n* = 20), inactive destructive (E ID; *n* = 20), active nondestructive (E AN; *n* = 20), or active destructive (E AD; *n* = 20) types of endometrosis. C group included category I (*n* = 20) samples; type E IN included categories IIa (*n* = 5), IIb (*n* = 11), and III (*n* = 4) samples; type E ID included categories IIa (*n* = 10), IIb (*n* = 5), and III (*n* = 5) samples; type E AN included categories IIa (*n* = 12), IIb (*n* = 5), and III (*n* = 3) samples; and type E AD included categories IIa (*n* = 3), IIb (*n* = 9), and III (*n* = 8) samples. Boxes represent lower quartile, median, and upper quartile, whereas whiskers represent minimum and maximum values. The dashed line separates the unaffected endometrium and types of affected endometrium. Lowercase letters indicate differences between histopathological types of endometrosis for *p* < 0.05.

Regarding histopathological types of equine endometrosis, the transcription was higher in the control group than in endometrosis types E ID and E AD for *ESR1* (*p* < 0.0001; Figure 2A), *ESR2* (*p* < 0.0001; Figure 2B), and *PGR* (*p* < 0.0001; Figure 2C). The expression of *ESR1* (*p* < 0.05) was higher in the control group than in endometrosis types E IN and E AN, but not *ESR2* and *PGR*. No differences were found between endometrosis types in the affected endometrium for all three studied receptors.

### 2.2. Phases of the Estrous Cycle and Ovarian Steroid Receptors Regarding Equine Endometrosis Categories

**Figure 3:**
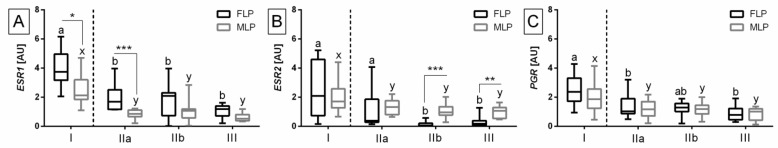
The transcription levels of (**A**) estrogen receptor α (*ESR1*), (**B**) estrogen receptor ß (*ESR2*), and (**C**) progesterone receptor (*PGR*) in the mares’ endometria in follicular (FLP; *n* = 50) or mid-luteal (MLP; *n* = 50) phases of the estrous cycle. The endometrial samples were classified as Kenney and Doig’s category I (*n* = 20), IIa (*n* = 30), IIb (*n* = 30), or III (*n* = 20). Category I included C group (*n* = 20) samples; category IIa included types E IN (*n* = 5), E ID (*n* = 10), E AN (*n* = 12), and E AD (*n* = 3) samples; category IIb included types E IN (*n* = 11), E ID (*n* = 5), E AN (*n* = 5), and E AD (*n* = 9) samples; and category III included types E IN (*n* = 4), E ID (*n* = 5), E AN (*n* = 3), and E AD (*n* = 8) samples. Boxes represent lower quartile, median, and upper quartile, whereas whiskers represent minimum and maximum values. The dashed line separates the categories of unaffected and affected endometria. Lowercase letters indicate differences between categories of endometrosis for *p* < 0.05. Asterisks indicate differences between phases of estrous cycle (* *p* < 0.05; ** *p* < 0.01; *** *p* < 0.0001).

The transcription of the studied genes regarding categories of equine endometrosis differed between the phases of the estrous cycle in selected categories for *ESR1* (Figure 3A) and *ESR2* (Figure 3B), but not for *PGR* (Figure 3C). As such, the transcription of *ESR1* was higher in FLP than in MLP in categories I (*p* = 0.008) and IIa (*p* < 0.0001), but not in categories IIb (*p* = 0.07) and III (*p* = 0.06). Concerning comparisons between categories in the selected phases of estrous cycle, in both FLP (*p* < 0.0001) and MLP (*p* < 0.0001) the transcription of *ESR1* was higher in category I than in categories IIa, IIb, and III, with no differences between categories in the affected endometrium (Figure 3A).

In addition, the transcription of *ESR2* was lower in FLP than MLP in categories IIb (*p* < 0.0001) and III (*p* = 0.002), but not in categories I (*p* = 0.41) and IIa (*p* = 0.15). Concerning comparisons between categories in the selected phases of estrous cycle, in FLP the transcription of *ESR2* was higher in categories I and IIa than in categories IIb and III (*p* < 0.0001), whereas in MLP the transcription of *ESR2* was higher in category I than in categories IIa, IIb, and III (*p* = 0.004). No differences were found between categories I and IIa, as well as IIb and III in FLP, and neither between categories IIa, IIb, and III in MLP (Figure 3B).

Concerning comparisons between categories in the selected phases of the estrous cycle, in FLP the transcription of *PGR* was higher in category I than in categories IIa and III (*p* = 0.0007). Nevertheless, in MLP the transcription of *PGR* was higher in category I than in categories IIa, IIb, and III (*p* = 0.002). No differences were found between categories I and IIb, but also IIa, IIb, and III in FLP, and likewise between categories IIa, IIb, and III in MLP (Figure 3C).

### 2.3. Phases of the Estrous Cycle and Ovarian Steroid Receptors Regarding Histopathological Types of Equine Endometrosis

**Figure 4:**
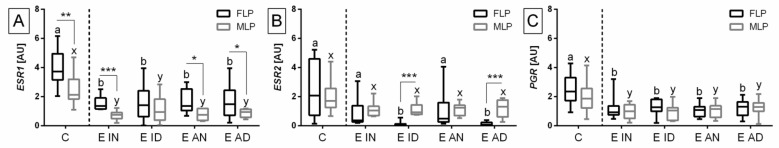
Transcription levels of (**A**) estrogen receptor α (*ESR1*), (**B**) estrogen receptor ß (*ESR2*), and (**C**) progesterone receptor (*PGR*) in the mares’ endometria in follicular (FLP; *n* = 50) or mid-luteal (MLP; *n* = 50) phases of the estrous cycle. The endometrial samples classified as control group (C; *n* = 20) or as inactive nondestructive (E IN; *n* = 20), inactive destructive (E ID; *n* = 20), active nondestructive (E AN; *n* = 20), or active destructive (E AD; *n* = 20) types of endometrosis. C group included category I (*n* = 20) samples; type E IN included categories IIa (*n* = 5), IIb (*n* = 11), and III (*n* = 4) samples; type E ID included categories IIa (*n* = 10), IIb (*n* = 5), and III (*n* = 5) samples; type E AN included categories IIa (*n* = 12), IIb (*n* = 5), and III (*n* = 3) samples; and type E AD included categories IIa (*n* = 3), IIb (*n* = 9), and III (*n* = 8) samples. Boxes represent lower quartile, median, and upper quartile, whereas whiskers represent minimum and maximum values. The dashed line separates the unaffected endometrium and types of affected endometrium. Lowercase letters indicate differences between histopathological types of endometrosis for *p* < 0.05. Asterisks indicate differences between phases of estrous cycle (* *p* < 0.05; ** *p* < 0.01; *** *p* < 0.0001).

The transcription of the studied genes regarding histopathological types of equine endometrosis differed between the phases of the estrous cycle in the selected endometrosis types for *ESR1* (Figure 4A) and *ESR2* (Figure 4B), but not for *PGR* (Figure 4C).

The levels of *ESR1* transcripts were higher in FLP than in MLP in the control group (*p* = 0.008) and endometrosis types E IN (*p* < 0.0001), E AN (*p* = 0.01), and E AD (*p* = 0.04), but not E ID (*p* = 0.43). Concerning comparisons between the histopathological types of endometrosis in the selected phases of estrous cycle, in both FLP (*p* = 0.001) and MLP (*p* = 0.0006) the transcription of *ESR1* was higher in the control group than in types E IN, E ID, E AN, and E AD, with no differences between types in affected endometrium (Figure 4A).

A decrease was observed in the transcription of *ESR2* in FLP, when compared to MLP in endometrosis types E ID (*p* < 0.0001) and E AD (*p* < 0.0001), but not in the control group (*p* = 0.41) and in E IN (*p* = 0.10) and E AN types (*p* = 0.35). Concerning the comparisons between histopathological types of endometrosis in the selected phases of the estrous cycle, in FLP the transcription of *ESR2* was higher in the control group and in endometrosis types E IN and E AN than in types E ID and E AD (*p* < 0.0001). In MLP, no differences were found in the transcripts of *ESR2* between the compared groups (*p* = 0.16). In addition, in the control group, mRNA levels did not differ from endometrosis types E IN, E AN, and between endometrosis types E ID and E AD in FLP (Figure 4B).

Concerning comparisons between endometrosis histopathological types in the selected phases of the estrous cycle, both in FLP (*p* = 0.005) and MLP (*p* = 0.007) the transcription of *PGR* was higher in the control group than in types E IN, E ID, E AN, and E AD. No differences were noticed between types in the affected endometrium both in FLP and MLP (Figure 4C).

### 2.4. The Nf-Κb Signaling Pathway and Ovarian Steroid Receptors in Endometrium Categories and Endometrosis Types

In category I, a strong negative correlation in MLP was noted between the transcripts of *ESR2* and IL-6 (ρ = −0.72; *p* = 0.01). In category IIa, positive correlations were observed between mRNA levels in both FLP and MLP. In FLP, a strong correlation between *PGR* and *RelA* (ρ = 0.75; *p* = 0.002) and a moderate correlation between *ESR2* and *HAS2* (ρ = 0.63; *p* = 0.01) were noted. In MLP, moderate correlations between *ESR1* and *IL-6* (ρ = 0.58; *p* = 0.03) and between *PGR* and *HAS2* (ρ = 0.61; *p* = 0.02) were found. Moreover, a moderate negative correlation in MLP was observed between the transcript levels of *PGR* and *NK-κB2* (ρ = −0.52; *p* = 0.04) (Table 1).

In category IIb, a moderate negative correlation in MLP was reported between transcriptions of *PGR* and *HAS3* (ρ = −0.64; *p* = 0.006). In category III, moderate negative correlations were observed in FLP for transcriptions of *ESR2* and *HAS1* (ρ = −0.69; *p* = 0.03), *ESR2* and *HAS3* (ρ = −0.67; *p* = 0.008), *PGR* and *HAS1* (ρ = −0.46; *p* = 0.04), and *PGR* and *HAS3* (ρ = −0.64; *p* = 0.03). Moreover, a moderate negative correlation in MLP was noted between transcriptions of *PGR* and *HAS1* (ρ = −0.66; *p* = 0.04). Concerning categories of equine endometrosis, the other values of Spearman’s correlation coefficient were not statistically significant (*p* > 0.05) (Table 1).

Regarding the histopathological types of equine endometrosis, in the inactive nondestructive type of endometrosis, a strong positive correlation in FLP was noted between transcriptions of *PGR* and *NK-κB2* (ρ = 0.77; *p* = 0.01). Moreover, moderate negative correlations were reported in MLP for transcriptions of *ESR2* and *RelA* (ρ = −0.66; *p* = 0.02), *ESR2* and *MCP-1* (ρ = −0.65; *p* = 0.04), *PGR* and *IL-6* (ρ = −0.66; *p* = 0.04), and *PGR* and *HAS2* (ρ = −0.65; *p* = 0.007). In the inactive destructive type of endometrosis, positive correlations were observed in FLP between transcripts of *ESR1* and *HAS1* (ρ = 0.76; *p* = 0.02) and *ESR1* and *HAS2* (ρ = 0.58; *p* = 0.04) as strong and moderate, respectively. Moreover, in MLP strong, moderate, and weak positive correlations were noted between transcriptions of *ESR1* and *IL-6* (ρ = 0.78; *p* = 0.004), *ESR1* and *RelA* (ρ = 0.53; *p* = 0.008), *ESR1* and *NK-κB1* (ρ = 0.40; *p* = 0.02), and *ESR1* and *MCP-1* (ρ = 0.39; *p* = 0.004), whereas strong and moderate negative correlations were observed between the mRNA levels of *ESR1* and *HAS1* (ρ = *−*0.78; *p* = 0.004) and *ESR1* and *HAS2* (ρ = −0.66; *p* = 0.02), respectively (Table 2).

In the active nondestructive type of endometrosis, in MLP, strong and moderate negative correlations were reported between transcriptions of *PGR* and *MCP-1* (ρ = −0.75; *p* = 0.01) and between *ESR2* and *NK-κB1* (ρ = −0.69; *p* = 0.03), respectively. A strong positive correlation was noted between transcriptions of *PGR* and *HAS3* (ρ = 0.75; *p* = 0.02). In the active destructive type of endometrosis, a strong positive correlation was observed in FLP between transcripts of *ESR1* and *HAS2* (ρ = 0.72; *p* = 0.02). Moreover, in FLP, strong and moderate negative correlations were reported between transcripts of *PGR* and *NK-κB1* (ρ = −0.78; *p* = 0.03), *ESR2* and *RelA* (ρ = −0.77; *p* = 0.004), and *ESR2* and *NK-κB1* (ρ = −0.66; *p* = 0.01), respectively. No other Spearman’s correlation coefficient values were statistically significant (*p* > 0.05) (Table 2).

## 3. Discussion

In comparison to the healthy endometrium, the transcription levels of *ESR1*, *ESR2*, and *PGR* decreased with the severity of endometrial fibrosis, although for *ESR2* this decrease started at category IIb. The results regarding the endometrial *ESR1* and *PGR* mRNA contents agree with the findings of Hoffman et al. [13], who reported a decrease in the protein expression of ovarian steroid receptors with the fibrosis and the study of Lehmann et al. [14] which showed a reduction in staining intensity for ER-α and PR in the stromal cells present in fibrosis areas around endometrial glands, regardless of the endometrium category. Concerning the histopathological types of equine endometrosis, the transcription levels of *ESR1*, *ESR2*, and *PGR* were lower in inactive and active types of destructive endometrosis in comparison to the healthy endometrium. Moreover, the transcription level of *ESR1* decreased similarly in inactive and active types of nondestructive endometrosis, which is in agreement with previous findings in the fibrotic stroma [13,14]. In contradiction to the current findings, in the active nondestructive type of endometrosis, a predominant increase in the ER-α and PR expression was reported [13,14], even though only in the glandular epithelia that were not differentiated from the fibrotic stromal cells in the current study.

In addition to the previous knowledge of the putative influence of fibrosis on ER-α expression [13], the current results suggest a relationship between the destructiveness of fibrosis and both ER-α and ER-ß gene transcription. Nevertheless, the precise kinetics and chronological succession of pathological events involving the increase in collagen deposition and impairment of ovarian steroid receptors in mare endometrosis remain unknown. It is worth noting that these data are in line with another study [13], where the most severe decrease in the expression of ER-α and PR was observed in the destructive fibrosis. As Hoffman et al. [13] and Lehamn et al. [14] did not investigate ER-ß expression, to the best of our knowledge this is the first report comparing the transcription level of *ESR2* between consecutive Kenney and Doig endometrial categories [15] and Hoffman et al. [13] histological types of equine endometrosis. Although in previous studies [13,14] an immunohistochemical examination was performed, in the present research, only the transcription analyses were considered. As the transcripts only partially explain the protein concentrations present in the tissue [42], and different processes can regulate mRNA and protein production and degradation [43], some differences between tissue and molecular expressions may be ascribed to post-transcriptional regulation.

It is well known that functional endometrial morphology, concerning proliferation and secretion, is consistent with the follicular and luteal phases of the estrous cycle [3,44]. Therefore, cell proliferation and apoptosis in the equine endometrium should be considered in two phases, stromal proliferation during the follicular phase, and epithelial proliferation during the luteal phase, both in healthy [23,45] and fibrotic endometria. However, the expression of ovarian steroid receptors in consecutive categories and histopathological types of equine endometrosis has not yet been compared between the phases of the estrous cycle at transcript levels.

In the unaffected endometrium, the transcription of *ESR1* was higher in FLP than in MLP in contrast to transcription of *PGR* and *ESR2*, which was similar in both phases. Although the transcription of *ESR1* observed in this study is consistent with work conducted by Silva et al. [21] and Hartt et al. [29], the *PGR* results are partially different. Likewise, regarding the transcription of *ESR1*, some studies reported that *PGR* transcription was higher in FLP than in MLP [29,46,47]. However, no differences in *PGR* mRNA levels between phases were found in the present study and others [21]. Moreover, our data on *ESR2* transcription are convergent with Silva et al. report [21], which is one of the few studies investigating the estrous cycle’s influence on *ESR2* expression in equine endometrium. Some discrepancies in the previous and current results may be explained by differences in the methodology applied herein, and in the previous studies [21,29,46,47,48], of which Silva and coworkers’ [21] methodology was the most similar to the one presented here.

In comparison to the unaffected endometrium, the transcription levels of *ESR1*, *ESR2*, and *PGR* generally decreased with the severity of endometrial fibrosis in both phases of the estrous cycle. The only two exceptions were for *ESR2* in category IIa and *PGR* in category IIb, both in the FLP, but not in the MLP, where their transcription levels did not differ from category I. Moreover, the differences in transcription of *ESR1* between FLP and MLP, which were referred to be detectable in healthy [21,29] and mildly affected endometria, became undetectable in moderate and severe endometrosis. In contrast, the transcription of *ESR2* in mare endometrium that was not affected by the phases of the estrous cycle [21] was lower in FLP than in MLP in moderate and severe endometrosis. It might be suggested that the transcription of endometrial steroid receptors is upregulated by estradiol and downregulated by progesterone in equine healthy endometrium [23,24,29], which is prone to dysregulation in the course of endometrosis.

After assigning endometrial samples to the FLP or MLP group, a decrease in the transcription levels of *ESR1* and *PGR* was shown in all histopathological types of equine endometrosis, in comparison to healthy endometrium, in both phases of the estrous cycle. Our results are in line with Hoffman et al. [13] and Lehamn et al. [14] immunohistochemistry findings in stromal cells. The differences in the transcription of *ESR1* between FLP and MLP detectable in healthy endometrium were still present in both active types of endometrosis, and the inactive nondestructive type, in contrast to inactive destructive endometrosis. The absence of differences in the transcription of *PGR* in both healthy and qualitatively affected endometrium may support the previous hypothesis that ER-dependent rather than PR-dependent endometrial function deregulation may play a greater role in the pathogenesis of endometrosis [13]. Interestingly, the transcription of *ESR2* was lower in FLP than in MLP in both destructive types of endometrosis, which was not seen in the control group or any other histopathological types. One may note that the activation of ER-α and ER-ß has opposite effects on gene transcription [49]. Thus, ER-ß may downregulate ER functions, as ER-ß shows an inhibitory activity on ER-α transcriptional activity [50] by forming heterodimers with ER-α [51,52]. Therefore, it can be suggested the estrogen secreted during FLP interacts with both ER-α and ER-ß, causing the classic ER-α-dependent uterotrophic effects, and ER-ß-dependent hindering of ER-α effects [26]. In both destructive types of endometrosis, when *ESR2* transcription is significantly low, the ER-ß-dependent inhibitory activity might be reduced, and despite no increase in *ESR1* transcription, the final ER-α-dependent uterotrophic effects may be enhanced.

Based on previous [24] and current results, one may conclude that in both destructive types of endometrosis, the ER-α abundance in the endometrial stroma is the highest in FLP. With similar peripheral estrogen physiological concentrations, characteristic of the FLP, mare endometrium with a higher ER-α abundance showed a stronger tissue effect, with estrogen-dependent cellular proliferation, as reported in other species and tissues [27,53]. Such an activation may overinfluence stromal differentiation and induce stroma–epithelium interactions [24], the latter of which is disturbed in destructive endometrosis. In a healthy endometrium, the effect of ovarian steroids on the glandular epithelia is mediated by the surrounding stroma [14,54]. It is an intact basal lamina in the healthy endometrium that ensures the complex paracrine interactions between the epithelia and the underlying stroma [13,55], inhibiting the direct interaction between stromal cells, epithelial cells, and the extracellular matrix (ECM). In destructive endometrosis, the damage of the basal lamina [5,13,56] allows for the direct interactions between epithelial cell surface integrins and the fibrotic ECM, and between stromal and epithelial cells [13]. Together with the altered ER-α and PR expression in epithelial cells [13,14], and the lowered *ESR2* transcription, they may become crucial for both stromal and epithelial cell integrity causing degeneration, endometrial glandular dilatation, and atypical differentiation of the affected glands [13,14], especially since *ESR2* transcription was similarly low in both moderate and severe endometrosis. The current estrous cycle-dependent results support Hoffman and collaborators’ [13] suggestion of advanced de-differentiation of the stromal cells within the fibrotic foci. However, they also put forward a contradictory statement that fibrotic stromal cells are unable to react to cyclic endocrine changes and become independent of hormonal regulation. The present data suggest that in endometrosis, the histological features of maldifferentiated stromal and glandular epithelia are asynchronous to the estrous cycle phase, which supports previous research [13]. However, it suggests that the endometrium becomes independent of estrous cycle regulation [13], whereas our study indicates that the asynchrony of the endometrium may be still estrous cycle-dependent but might be caused by a deregulation of ER functions related to the impairment of ER-α and ER-ß activity.

In a previous study, Rebordão et al. [57] also suggested that the pathogenesis of equine endometrosis might be somehow connected with the estrous cycle. This statement was previously supported by our previous reports, where the transcription of NF-κB pathway genes regarding Kenney and Doig’s endometrial categories [31] and the histopathological types of endometrosis [32] were investigated. The NF-κB is known for stimulating ECM deposition in various tissues [33,34,37,38,58] and mediating hyaluronan synthesis by fibroblasts [59]. Therefore, the NF-κB-dependent activation of proinflammatory molecules may play a role in gland deformation and damage and altered interactions between epithelial cells and the fibrotic ECM [31,32]. As the histopathological type of endometrosis depicted a larger modification of ECM, especially the increase in proteoglycans, fibronectin, and laminin expression [13,15], we have suggested the active remodeling of ECM in FLP. With the evolution of the concepts of equine endometrosis, it was shown that the ECM architecture and endometrial function are affected by the neutrophil extracellular traps (NETs), pro-inflammatory cytokines, pro-fibrotic pathways, growth factors and epigenetics [12].

In our previous study, just in FLP equine endometrium, the transcriptions of *RelA*, *NF-κB1*, *IL-6*, and *HAS2* were higher in the active destructive type of endometrosis than in the control group [31]. In the current study, we supported these findings with the strong and moderate negative correlations reported just in E AD, for the transcription between *ESR2* and *RelA* or *NF-κB1*, respectively, as well as the strong correlation for the transcription between *PGR* and *RelA*, and between *ESR1* and *HAS2*, negative and positive, respectively. Moreover, in MLP an increase in *RelA*, *NF-κB1*, and *MCP-1* transcription was noted in E ID [32], which in the current study is supported by the weak to moderate positive correlations with *ESR1* transcription. Concerning Kenney and Doig’s categories of endometrosis [15], in our previous study *RelA*, *NF-κB1*, *NF-κB2*, *HAS1*, and *HAS3* transcriptions increased similarly with the degree of endometrial fibrosis, just in FLP [31], which in the current study is supported by the correlation with *ESR2* and *PGR* transcription but only for *HAS1* and *HAS3*. Although these results support the previous argument that the endometrial fibrotic changes mediated by the canonical NF-κB pathway are estrous cycle-dependent [31,32], the direction of these relationships and co-localization of corresponding protein concentrations in the tissue require further research. However, it can be suggested that the metabolic activity of fibroblasts in the equine endometrium affected by endometrosis may also depend on deregulation by ovarian steroid hormones. Since NF-κB inhibitors have been successfully studied in suppressing ECM deposition in various tissues [60,61,62], further works are required to establish the clinical applicability of research data on possible specific treatment of equine endometrosis.

## 4. Materials and Methods

### 4.1. Biological Material Collection

In the current study, the biological material consisted of equine internal genitalia and blood collected from 100 Polish warmblood mares (aged from 3 to 25 years) at a commercial abattoir in Poland. Biological sample collection was performed post-mortem, which does not fall under the legislation for the protection of animals used for scientific purposes, national decree-law (Dz. U. 2015 poz. 266) and EU law (2010-63-EU directive). Thus, no Ethical Committee’s permission was needed for sample retrieval after slaughter (decision of II Local Committee for Ethics in Animal Research WULS in Warsaw from 27 October 2021). Samples were obtained in the reproductive season from April to September.

Endometrial samples with a minimal size of 10 × 5 × 5 mm were collected from the junction between the uterine body and one uterine horn. Samples were collected immediately after evisceration. All samples were immersed in appropriate solutions, as follows: ovaries in cold 0.9% NaCl (Polfa S.A., Lublin, Poland); one endometrial sample into RNase-free Eppendorf tubes (Eppendorf AG, Hamburg, Germany) and immediately snap-frozen in liquid nitrogen, and another endometrial sample into 10% neutral phosphate-buffered formalin (Sigma-Aldrich, Poznan, Poland). Then, samples were transported to the laboratory under specific conditions: ovaries at +4 °C, endometrial samples for gene transcription analyses in liquid nitrogen, and endometrial samples for histological examination at room temperature. In the laboratory, ovaries were sectioned and the presence and diameter of follicles and/or corpus luteum were noted. Endometrial samples for gene transcription analyses were stored at −80 °C, whereas endometrial samples for histological examination were fixed in formalin for 24 h, moved to 70% ethanol (Sigma-Aldrich, Poznan, Poland) for one week at room temperature, and then embedded in paraffin (Sigma-Aldrich, Poznan, Poland) for standard histological staining procedures.

Blood samples were collected into dry tubes (BD Vacutainer^®^, Plymouth, UK), transported to the laboratory at +4 °C, and centrifuged (2000× *g*, 5 min). The serum, free from any apparent hemolysis, was aspirated and stored at −20 °C.

### 4.2. Phases of Estrous Cycle Determination

The phases of the estrous cycle were determined based on the concentration of ovarian steroid hormones, 17 ß-estradiol (E_2_) and progesterone (P_4_), as well as on the macroscopic examination of mares’ ovaries, according to da Costa et al. protocol [45]. The concentrations of E_2_ and P_4_ were determined by commercial radioimmunoassay with a sensitivity 1.36 pg/mL (curve range 2.52 pg/mL to 22.8 pg/mL) for E_2_ (Estrus-Us-Ct, Cis Bioassays, Codolet, France) with intra-assay coefficient of variation <6.9% and inter-assay coefficient of variation <9.1%; and 0.05 ng/mL (curve range 0.12 ng/mL to 18.38 ng/mL) for P_4_ with intra-assay coefficient of variation <5.6% and inter-assay coefficient of variation <8.8% (KIP 1458; DIAsource ImmunoAssays SA, Ottignies-Louvain-la-Neuve, Belgium). Mares were assigned to the mid-luteal phase group (MLP; *n* = 50) when serum concentrations of E_2_ and P_4_ were <4 pg/mL and >1 ng/mL, respectively; and on both ovaries, none of the follicles >35 mm in diameter and at least one corpus luteum was demonstrated. Mares were included into the follicular phase group (FLP; *n* = 50) when serum concentrations of E_2_ and P_4_ were >4 pg/mL and <1 ng/mL, respectively; and there was at least one follicle >35 mm in diameter in any of the ovaries and no corpus luteum.

### 4.3. Histopathological Examination of Mares’ Endometria

Formaldehyde was flushed from samples with 70% ethyl alcohol. Samples were paraffined with standard protocols and cut into 6 μm sections with rotation microtome Leica RM2255 (Kawa-Ska, Zalesie Gorne, Polska) and mounted on glass slides. Slides were deparaffinized and rehydrated in a series of immersions in xylene and decreasing concentrations of ethanol (Sigma-Aldrich, Poznan, Poland). Then, samples were stained using standard hematoxylin-eosin (HE) protocol (hematoxylin, 3801520E, Leica, Buffalo Grove, IL, USA; eosin, HT1103128; SigmaAldrich, Poznan, Poland) and mounted under Canadian balsam resin for histological evaluation (Sigma-Aldrich, Poznan, Poland). The HE-stained slides were evaluated under a light microscope (Olympus BX43, Warsaw, Poland, magnification 40×–1000×). The presence of inflammation and the appearance or severity of pathological degenerative changes were microscopically assessed. For RNA isolation, only samples that did not appear actively inflamed in the macroscopic examination and did not reveal any inflammatory cell infiltration in the histopathological examination were selected.

Equine endometrosis was recognized when the microscopic hallmark, the concentric arrangement of stromal cells and/or collagen fibers around affected glands, was observed [3,63]. The same endometrial samples (*n* = 100) were independently classified twice, (i) as belonging to category I, IIa, IIb, or III of Kenney and Doig [15] according to the degree of fibrosis [15], and (ii) as belonging to histopathological type E IN,E ID,E AN, or E AD of Hoffmann and collaborators [13] according to specific pathological features [13].

The mares with healthy endometrial tissue were included in the category I (category I; *n* = 20) of (i) Kenney and Doig’s classification and in the control group (C; *n* = 20) of (ii) Hoffmann and collaborators’ classification. Whenever endometrosis was present, samples were further graded regarding (i) Kenney and Doig’s categories and (ii) Hoffmann and collaborators’ histopathological types. Thus, the remaining 80 endometrial samples were categorized twice. Firstly, endometrial samples (*n* = 80) were assigned to remaining the three Kenney and Doig’s categories, as follows: (i) mild endometrosis (category IIa; *n* = 30), (ii) moderate endometrosis (category IIb; *n* = 30), and (iii) severe endometrosis (category III; *n* = 20). Secondly, endometrial samples (*n* = 80) were assigned to each of the four Hoffmann and collaborators’ histopathological types, as follows: (i) inactive-nondestructive endometrosis (E IN; *n* = 20), (ii) inactive-destructive endometrosis (E ID; *n* = 20), (iii) active-nondestructive endometrosis (E AN; *n* = 20), and (iv) active-destructive endometrosis (E AD; *n* = 20).

A detailed distribution of samples between the two classification systems is presented in Table 3.

Concerning Kenney and Doig’s classification, category IIa included types E IN (*n* = 5), E ID (*n* = 10), E AN (*n* = 12), and E AD (*n* = 3) samples; category IIb included types E IN (*n* = 11), E ID (*n* = 5), E AN (*n* = 5), and E AD (*n* = 9) samples; and category III included types E IN (*n* = 4), E ID (*n* = 5), E AN (*n* = 3), and E AD (*n* = 8) samples.

Concerning Hoffmann and collaborators’ classification, type E IN included categories IIa (*n* = 5), IIb (*n* = 11), and III (*n* = 4) samples; type E ID included categories IIa (*n* = 10), IIb (*n* = 5), and III (*n* = 5) samples; type E AN categories included IIa (*n* = 12), IIb (*n* = 5), and III (*n* = 3) samples; and type E AD included categories IIa (*n* = 3), IIb (*n* = 9), and III (*n* = 8) samples.

In all groups representing the categories and histopathological types of equine endometrosis, half of the samples were collected from mares in FLP (category I/C, *n* = 10; category IIa, *n* = 15; category IIb, *n* = 15; category III, *n* = 10; E IN, *n* = 10; E ID, *n* = 10; E AN, *n* = 10; E AD, *n* = 10) and the other half in MLP (category I/C, *n* = 10; category IIa, *n* = 15; category IIb, *n* = 15; category III, *n* = 10; E IN, *n* = 10; E ID, *n* = 10; E AN, *n* = 10; E AD, *n* = 10). In the same endometria, the transcription of selected genes involved in the NF-κB signaling pathway (*RelA*; *NK-κB1*; *NK-κB2*; *MCP-1*; *IL-6*; *HAS1*; *HAS2*; *HAS3*) was investigated. However, these whole results have been previously reported for both, categories [31] and histological types of equine endometrosis [32]. The raw data of the transcription of genes investigated in the present study were used in a previous one to find the putative relationship between transcription of ovarian steroid receptor genes and the NF-κB-dependent signaling pathway in the mare’s endometrium affected by equine endometriosis.

### 4.4. Gene Transcription Evaluation in Mares’ Endometria

Endometrial samples stored at −80 °C were mechanically disrupted in a liquid nitrogen environment. Then, 50 mg of each sample were homogenized in Lysing Matrix D tubes (MP Biomedicals, Irvine, CA, USA), and total RNA was extracted using High Pure RNA Tissue Kit (Roche, Rotkreuz, Switzerland) using a standard protocol. Afterward, a DNase treatment was performed. The RNA concentration was determined using DS-11 FX spectrophotometer (DeNovix, Wilmington, DE, USA) with absorbance ratios A260/280 and A260/230 of approximately 2.0. Further analysis was allowed only for these samples that have RNA content above 100 ng. None of the samples was excluded due to insufficient RNA content.

Real-time PCR (qPCR) amplification was performed using a TaqMan™ RNAto-CT™ 1-Step Kit (No 4392938, ThermoFisher, Swedesboro, NJ, USA) and a Quant-Studio™ 6 Flex Real-Time PCR System (Applied Biosystems, Wilmington, DE, USA). The commercially available equine-specific TaqMan Gene Expression Assays (No 4448892 and 4441114, ThermoFisher, Swedesboro, NJ, USA) were used. Primers specific for the selected transcripts *ESR1*, *ESR2*, and *PGR*, were designed using Primer-BLAST (NCBI; Table 4). Two reference genes, *GAPDH* (Ec03210916_gH) and *HPRT1* (Ec03470217_m1), were also evaluated. Real-time PCR reaction had a 10 mL volume and included 15 ng of total RNA, 5 mL of TaqMan^®^ RTPCR Mix (2×), 0.25 mL of TaqMan^®^ RT Enzyme Mix (40×), 0.5 mL of TaqMan probe, and both PCR primers (ThermoFisher, Swedesboro, NJ, USA) for each gene of interest. The PCR protocol included four steps, as follows: reverse transcription (15 min at 48 °C), enzyme activation (10 min at 95 °C), 40 cycles of denaturation (15 s at 95 °C), and annealing/extension (1 min at 60 °C). Each sample was run in duplicate [31,32].

In each endometrial sample, transcription of the three studied genes (*ESR1*, *ESR2*, and *PGR*) and two independent endogenous reference genes (*GAPDH* and *HPRT1*) was assessed by qPCR. Each endometrial sample was triply categorized using estrous cycle criterion and two endometrosis criteria (the Kenney and Doig’s classification and histopathological types of equine endometrosis defined by Hoffman and collaborators). Raw data of genes transcription were normalized using the geometric mean of mRNA detected from two reference genes. The semi-quantitation of the target gene expression was performed in a comparative CT method (ΔΔCT method), where the target gene expression in the samples of category I/group C was considered as ΔCt Control Value [31,32].

### 4.5. Statistical Analysis

Statistical analysis was performed using GraphPad Prism6 software (GraphPad Software Inc., San Diego, CA, USA). Data analysis was performed in the following three steps: (i) testing the differences between categories and histopathological types without considering the phase of the estrous cycle; (ii) testing the differences between categories, histopathological types, and phases considering the phase of the estrous cycle; (iii) calculating the correlation coefficient between transcription of genes of the ovarian steroid receptors and the NF-κB-dependent signaling pathway.

Data from 100 endometrial samples were presented as separate data series of Expression Fold Change (2^−ΔΔCt^) of the qPCR *ESR1*, *ESR2*, and *PGR* data for each studied category and histopathological type of equine endometrosis. Data series were tested independently for univariate distributions using a Shapiro–Wilk normality test. As at least one data series showed a non-Gaussian distribution, the comparisons between data series were assessed using the Kruskal–Wallis test followed by the Dunn’s multiple comparisons test, independently from the studied categories and histopathological types. The significance level was established as *p* < 0.05.

Then, each studied category and histopathological type was divided into FLP and MLP data series, and a Shapiro–Wilk normality test was performed for each new data series. As at least one data series showed a non-Gaussian distribution, the comparisons between data series were assessed using the Kruskal–Wallis test followed by the Dunn’s multiple comparisons test, independently from the studied categories in FLP and MLP, as well as histopathological types in FLP and MLP. The comparisons between the phases of the estrous cycle were performed by Unpaired t-test with Welch’s correction for Gaussian data pairs or Mann–Whitney test for non-Gaussian data pairs. For both tests, the significance level was established as *p* < 0.05.

Spearman’s rank correlation coefficient (ρ) was calculated for all pairs of (iii) data series represented *ESR1*, *ESR2*, and *PGR* as well *RelA*, *NK-κB1*, *NK-κB2*, *MCP-1*, *IL-6*, *HAS1*, *HAS2*, and *HAS3*, for FLP and MLP separately. The value of ρ reflected the consistency when the P was considered significant (*p* < 0.05).

Numerical data in box plots are represented by minimum and maximum values, lower and upper quartiles as well as medians. Numerical data in tables are reported as ρ; ρ in each cell for FLP; MLP, together. All statistical analyses were performed using the GraphPad Prism6 software (GraphPad Software Inc., San Diego, CA, USA).

## 5. Conclusions

In comparison to the unaffected endometrium, the transcription of *ESR1*, *ESR2*, and *PGR* decreased with the severity of endometrial fibrosis as well as in inactive and active types of destructive endometrosis. In addition, when the effect of the phases of the estrous cycle was considered, differences in the transcription of *ESR1*, *ESR2*, and *PGR* between phases were observed in the specific categories and histopathological type of equine endometrosis. The transcription of endometrial steroid receptors is subject to dysregulation with the severity of equine endometrosis, especially in both destructive types of endometrosis. Moreover, the ER-dependent rather than PR-dependent deregulation seems to play a greater role in the pathogenesis of endometrosis. Therefore, the role of the so far not assessed *ESR2* should be investigated. It is worth noting that both in FLP and MLP specific moderate and strong correlations between *ESR1*, *ESR2*, *PGR* and *RelA*, *NK-κB1*, *NK-κB2*, *MCP-1*, *IL-6*, *HAS1*, *HAS2*, and *HAS3* were evidenced, especially in both types of destructive endometrosis. Thus, the previous thesis that the endometrial fibrotic changes mediated by the canonical NF-κB pathway are estrous cycle-dependent was upheld. However, the specific relationship and co-localization of corresponding proteins in the endometrial tissue require further research.

## Figures and Tables

**Table 1:** Spearman’s correlation coefficient (ρ) between transcription levels of ovarian steroid receptors (*ESR1*, *ESR2*, *PGR*) and transcription levels of selected proteins of the nuclear factor κB (NF-κB) signaling pathway (*RelA*, *NK-κB1*, *NK-κB2*, *MCP-1*, *IL-6*, *HAS1*, *HAS2*, *HAS3*) in equine endometrium classified according to different categories (I, IIa, IIb, III). The value of ρ is reported for the follicular (FLP) and mid-luteal (MLP) phases of the estrous cycle. In one cell in the table, both ρ values are reported as ρ for FLP; ρ for MLP.

	*RelA*	*NK-* *κB1*	*NK-* *κB2*	*MCP-1*	*IL-6*	*HAS1*	*HAS2*	*HAS3*
Category I								
*ESR1*	0.14; 0.17	0.00; −0.12	0.01; −0.31	−0.15; 0.37	0.09; −0.21	0.00; −0.47	−0.05; 0.13	−0.09; 0.24
*ESR2*	0.05; 0.17	−0.05; 0.49	−0.09; −0.14	−0.10; 0.46	0.40; −0.75 *	0.63; 0.43	−0.05; −0.32	−0.40; −0.03
*PGR*	−0.28; 0.04	0.10; 0.49	0.06; 0.39	−0.34; −0.19	−0.18; 0.04	−0.09; 0.41	0.10; 0.02	0.18; −0.28
Category IIa								
*ESR1*	0.36; 0.18	−0.01; 0.35	0.30; −0.28	−0.22; 0.39	−0.22; 0.58 *	0.20; 0.05	0.41; −0.06	0.11; 0.41
*ESR2*	0.37; 0.12	0.12; −0.02	0.20; −0.19	−0.47; 0.19	−0.47; 0.05	0.17; 0.21	0.63 *; 0.09	0.38; 0.39
*PGR*	0.75 *; −0.24	−0.04; −0.05	0.35; −0.52 *	−0.15; 0.41	−0.15; 0.28	0.20; 0.17	0.37; 0.61 *	0.27; 0.16
Category IIb								
*ESR1*	0.48; 0.27	−0.09; −0.09	0.19; −0.16	−0.07; 0.03	−0.30; −0.24	−0.30; 0.03	0.03; 0.29	−0.48; 0.38
*ESR2*	0.28; 0.22	−0.02; 0.43	0.11; 0.32	−0.05; −0.05	−0.22; 0.01	−0.14; −0.02	−0.26; −0.02	−0.13; 0.02
*PGR*	0.30; −0.34	0.04; −0.11	0.14; −0.03	0.00; −0.34	−0.20; 0.25	−0.20; −0.17	−0.13; −0.03	−0.16; −0.64 *
Category III								
*ESR1*	−0.16; 0.00	−0.09; 0.48	−0.15; 0.43	0.07; 0.16	0.02; 0.08	−0.16; 0.28	0.01; −0.10	−0.14; −0.07
*ESR2*	−0.18; −0.39	−0.04; −0.09	0.15; 0.10	−0.17; 0.10	−0.01; 0.46	−0.69 *; 0.21	−0.44; 0.15	−0.67 *; −0.16
*PGR*	−0.40; 0.27	−0.14; 0.09	−0.34; 0.22	−0.45; −0.32	0.31; −0.21	−0.46 *; −0.66 *	−0.69; −0.05	−0.64 *; 0.45

*ESR1—*estrogen receptor α gene; *ESR2—*estrogen receptor ß gene; *PGR*—progesterone receptor gene; *RelA*—the nuclear factor κB subunit RelA; *NK-κB1*—the nuclear factor κB subunit 1; *NK-κB2*—the nuclear factor κB subunit 2; *MCP-1*—monocyte chemoattractant protein-1 gene; *IL-6*—interleukin-6; *HAS1*—hyaluronan synthase 1 gene; *HAS2*—hyaluronan synthase 2 gene; *HAS3*—hyaluronan synthase 3 gene. Bolded values of ρ and asterisk reflect consistency between selected transcript levels for *p* < 0.05.

**Table 2:** Spearman’s correlation coefficient (ρ) between transcription levels of ovarian steroid receptors (*ESR1*, *ESR2*, *PGR*) and transcription levels of selected proteins of the nuclear factor κB (NF-κB) signaling pathway (*RelA*, *NK-κB1*, *NK-κB2*, *MCP-1*, *IL-6*, *HAS1*, *HAS2*, *HAS3*) in equine endometrium classified according to different histopathological types of endometrosis (E IN, E ID, E AN, E AD). The value of ρ is reported for the follicular (FLP) and mid-luteal (MLP) phases of the estrous cycle. In one cell in the table, both ρ values are reported as ρ for FLP; ρ for MLP.

	*RelA*	*NK-* *κB1*	*NK-* *κB2*	*MCP-1*	*IL-6*	*HAS1*	*HAS2*	*HAS3*
E IN								
*ESR1*	0.12; −0.21	−0.06; 0.36	0.58; 0.18	−0.12; −0.12	0.07; 0.22	0.11; 0.45	−0.50; −0.33	−0.34; 0.56
*ESR2*	0.14; −0.66 *	−0.43; −0.62	0.60; −0.14	0.10; −0.65 *	0.29; 0.02	0.35; 0.12	−0.43; 0.53	0.34; −0.05
*PGR*	0.09; 0.16	−0.23; 0.31	0.77 *; −0.11	−0.21; −0.15	0.04; −0.66 *	0.09; 0.05	0.37; −0.65 *	0.00; 0.35
E ID								
*ESR1*	−0.55; 0.53 *	−0.27; 0.40 *	−0.18; −0.66	−0.18; 0.39 *	−0.18; 0.78 *	0.76 *; −0.78 *	0.58 *; −0.66 *	0.58; −0.12
*ESR2*	0.51; 0.19	0.38; 0.14	0.45; 0.66	0.45; −0.36	0.45; 0.55	−0.34; 0.55	−0.11; 0.66	0.11; 0.64
*PGR*	−0.51; 0.00	−0.06; 0.54	−0.13; 0.03	−0.13; 0.27	−0.13; 0.32	0.27; 0.32	−0.13; 0.03	0.13; −0.27
E AN								
*ESR1*	−0.12; −0.30	0.31; 0.00	−0.31; −0.34	0.39; 0.33	−0.31; 0.31	−0.39; −0.11	0.39; −0.12	−0.03; −0.33
*ESR2*	−0.21; 0.17	0.27; −0.69 *	−0.27; −0.48	0.44; 0.10	−0.27; 0.22	−0.44; −0.03	0.44; 0.50	0.03; −0.10
*PGR*	0.53; 0.06	0.18; 0.47	−0.18; −0.21	−0.37; −0.75 *	−0.18; 0.25	0.37; 0.20	−0.37; 0.31	−0.45; 0.75 *
E AD								
*ESR1*	0.23; −0.23	−0.41; 0.36	0.19; −0.07	−0.21; 0.03	−0.37; 0.15	0.10; −0.16	0.72 *; 0.06	0.19; 0.48
*ESR2*	−0.77 *; 0.02	−0.66 *; −0.13	−0.23; 0.54	0.42; −0.09 *	−0.11; 0.72	−0.31; −0.43	0.15; −0.06	−0.15; −0.10
*PGR*	−0.20; 0.05	−0.78 *; 0.15	0.36; 0.10	−0.07; −0.01	−0.51; 0.04	0.10; 0.00	−0.02; −0.06	0.28; 0.42

*ESR1—*estrogen receptor α gene; *ESR2—*estrogen receptor ß gene; *PGR*—progesterone receptor gene; *RelA*—the nuclear factor κB subunit RelA; *NK-κB1*—the nuclear factor κB subunit 1; *NK-κB2*—the nuclear factor κB subunit 2; *MCP-1*—monocyte chemoattractant protein-1 gene; *IL-6*—interleukin-6; *HAS1*—hyaluronan synthase 1 gene; *HAS2*—hyaluronan synthase 2 gene; *HAS3*—hyaluronan synthase 3 gene. E IN—type inactive nondestructive of endometrosis; E ID—type inactive destructive of endometrosis; E AN—type active nondestructive of endometrosis; E AD—type active destructive of endometrosis. Bolded values of ρ and asterisk reflect consistency between selected transcript levels for *p* < 0.05.

**Table 3:** The distribution of samples between the two classification systems of (i) Kenney and Doig and (ii) Hoffmann and collaborators.

	C/I	IIa	IIb	III	Totals
E IN		5	11	4	20
E ID		10	5	5	20
E AN		12	5	3	20
E AD		3	9	8	20
Totals	20	30	30	20	100/80

Kenney and Doig’s classification: I—category I; IIa—category IIa; IIb—category IIb; III—category III; Hoffmann and collaborators’s classification: C—control group; E IN—type inactive nondestructive of endometrosis; E ID—type inactive destructive of endometrosis; E AN—type active nondestructive of endometrosis; E AD—type active destructive of endometrosis.

**Table 4:** Forward and reverse primer sequences used for estrogen receptor α (*ESR1*) and estrogen receptor ß (*ESR2*), and progesterone receptor (*PGR*).

Gene	Primer Sequence
*ESR1*	Forward: 5′-TCCATGGAGCACCCAGGAAAGC-3′ Reverse: 3′-CGGAGCCGAGATGACGTAGCC-5′
*ESR2*	Forward: 5′-TCCTGAATGCTGTGACCGAC-3′ Reverse: 3′-GTGCCTGACGTGAGAAAGGA-5′
*PGR*	Forward: 5′-CTTCCCCGACTGCGCGTACC-3′ Reverse: 3′-TTGTGTGGCTGGAAGTCGCCG-5′

## Data Availability

The data presented in this study are available on request from the corresponding author.

## References

[1] Aupperle H., Schoon D., Schoon H.-A. (2004). Physiological and pathological expression of intermediate filaments in the equine endometrium. Res. Vet. Sci..

[2] Da Costa R.R., Ferreira-Dias G., Mateus L., Korzekwa A., Andronowska A., Platek R., Skarzynski D.J. (2007). Endometrial nitric oxide production and nitric oxide synthases in the equine endometrium: Relationship with microvascular density during the estrous cycle. Domest. Anim. Endocrinol..

[3] Schöniger S., Schoon H.A. (2020). The healthy and diseased equine endometrium: A review of morphological features and molecular analyses. Animals.

[4] Skarzynski D.J., Szóstek-Mioduchowska A.Z., Rebordão M.R., Jalali B.M., Piotrowska-Tomala K.K., Leciejewska N., Łazarczyk M., Ferreira-Dias G.M. (2020). Neutrophils, monocytes and other immune components in the equine endometrium: Friends or foes?. Theriogenology.

[5] Hoffmann C., Bazer F.W., Klug J., Aupperle (2009). H.; Ellenberger, C.; Schoon, H.A. Immunohistochemical and histochemical identification of proteins and carbohydrates in the equine endometrium Expression patterns for mares suffering from endometrosis. Theriogenology.

[6] Rebordão M.R., Galvão A., Szóstek A., Amaral A., Mateus L., Skarzynski D.J., Ferreira-Dias G. (2014). Physiopathologic mechanisms involved in mare endometrosis. Reprod. Dom. Anim..

[7] Szóstek-Mioduchowska A., Leciejewska N., Zelmańska B., Staszkiewicz-Chodor J., Ferreira-Dias G., Skarzynski D. (2020). Lysophosphatidic acid as a regulator of endometrial connective tissue growth factor and prostaglandin secretion during estrous cycle and endometrosis in the mare. BMC Vet. Res..

[8] Minkwitz C., Schoon H.-A., Zhang Q., Schöniger S. (2019). Plasticity of endometrial epithelial and stromal cells—A new approach towards the pathogenesis of equine endometrosis. Reprod. Domest. Anim..

[9] Szóstek-Mioduchowska A.Z., Lukasik K., Skarzynski D.J., Okuda K. (2019). Effect of transforming growth factor-β1 on α-smooth muscle actin and collagen expression in equine endometrial fibroblasts. Theriogenology.

[10] Mambelli L.I., Mattos R.C., Winter G.H.Z., Madeiro D.S., Morais B.P., Malschitzky E., Miglino M.A., Kerkis A., Kerkis I. (2014). Changes in expression pattern of selected endometrial proteins following mesenchymal stem cells infusion in mares with endometrosis. PLoS ONE.

[11] Amaral A., Fernandes C., Morazzo S., Rebordão M.R., Szóstek-Mioduchowska A., Lukasik K., Gawronska-Kozak B., da Gama L.T., Skarzynski D.J., Ferreira-Dias G. (2020). The inhibition of cathepsin G on endometrial explants with endometrosis in the mare. Front. Vet. Sci..

[12] Katila T., Ferreira-Dias G. (2022). Evolution of the Concepts of Endometrosis, Post Breeding Endometritis, and Susceptibility of Mares. Animals.

[13] Hoffman C., Ellenberger C., Mattos R.C., Aupperle H., Dhein S., Stief B., Schoon H.-A. (2009). The equine endometrosis: New insights into the pathogenesis. Anim. Reprod. Sci..

[14] Lehmann J., Ellenberger C., Hoffmann C., Bazer F.W., Klug J., Allen W.R., Sieme H., Schoon H.A. (2011). Morpho-functional studies regarding the fertility prognosis of mares suffering from equine endometrosis. Theriogenology.

[15] Kenney R.M., Doig P.A., Morrow D.A. (1986). Equine endometrial biopsy. Current Therapy in Theriogenology.

[16] Schoon H.-A., Wiegandt I., Schoon D., Aupperle H., Bartmann C.-P. (2000). Functional disturbances in the endometrium of barren mares: A histological and immunohistological study. J. Reprod. Fertil. Suppl..

[17] Brunckhorst D., Schoon H.A., Bader H., Sieme H. (1991). Morphologische, enzym- und immunhistochemische Charakteristika des endometrialen Zyklus bei der Stute. Fertilitat.

[18] Walter I., Klein M., Handler J., Aurich J.E., Reifinger M., Aurich C. (2001). Lectin binding patterns of uterine glands in mares with chronic endometrial degeneration. Am. J. Vet. Res..

[19] Wilkenfeld S.R., Lin C., Frigo D.E. (2018). Communication between genomic and non-genomic signaling events coordinate steroid hormone actions. Steroids.

[20] Enmark E., Pelto-Huikko M., Grandien K., Lagercrantz S., Lagercrantz J., Fried G., Nordenskjold M., Gustafsson J.A. (1997). Human estrogen receptor b-gene structure, chromosomal localization, and expression pattern. J. Clin. Endocr. Metab..

[21] Silva E.S.M., Scoggin K.E., Canisso I.F., Troedsson M.H.T., Squires E.L., Ball B.A. (2014). Expression of receptors for ovarian steroids and prostaglandin E2 in the endometrium and myometrium of mares during estrus, diestrus and early pregnancy. Anim. Reprod. Sci..

[22] Mote P.A., Arnett-Mansfield R.L., Gava N., Defazio A., Mulac-Jericevic B., Conneely O.M., Clarke C.L. (2006). Overlapping and distinct expression of progesterone receptors A and B in mouse uterus and mammary gland during the estrus cycle. Endocrinology.

[23] Watson E.D., Skolnik S.B., Zanecosky H.G. (1992). Progesterone and estrogen receptor distribution in the endometrium of the mare. Theriogenology.

[24] Aupperle H., Özgen S., Schoon H.A., Schoon D., Hoppen H.O., Sieme H., Tannapfel A. (2000). Cyclical endometrial steroid hormone receptor expression and proliferation intensity in the mare. Equine Vet. J..

[25] DeFranco D.B. (2002). Navigating steroid hormone receptors through the nuclear compartment. Mol. Endocrinol..

[26] Weihua Z., Saji S., Makine S., Cheng G., Jensen E.V., Warner M., Gustafsson J.A. (2000). Estrogen receptor (ER) ß, a modulator of ERα in the uterus. Proc. Natl. Acad. Sci. USA.

[27] Cunha G.R., Cooke P.S., Kurita T. (2004). Role of stromal-epithelial interactions in hormonal responses. Arch. Histol. Cytol..

[28] Mulac-Jericevic B., Lydon J.P., Demayo F.J., Conneely O.M. (2003). Defective mammary gland morphogenesis in mice lacking the progesterone receptor B isoform. Proc. Natl. Acad. Sci. USA.

[29] Hartt L.S., Carling S.J., Joyce M.M., Johnson G.A., Vanderwall D.K., Ott T.L. (2005). Temporal and spatial associations of oestrogen receptor alpha and progesterone receptor in the endometrium of cyclic and early pregnant mares. Reproduction.

[30] Cooke P.S., Buchanan D.L., Young P., Setiawan T., Brody J., Korach K.S., Taylor J., Lubahn D.B., Cunha G.R. (1997). Stromal estrogen receptors mediate mitogenic effects of estradiol on uterine epithelium. Proc. Natl. Acad. Sci. USA.

[31] Domino M., Jasinski T., Kautz E., Juszczuk-Kubiak E., Ferreira-Dias G., Zabielski R., Sady M., Gajewski Z. (2020). Expression of genes involved in the NF-κB-dependent pathway of the fibrosis in the mare endometrium. Theriogenology.

[32] Jasiński T., Zdrojkowski Ł., Kautz E., Juszczuk-Kubiak E., Ferreira-Dias G., Domino M. (2021). Equine Endometrosis Pathological Features: Are They Dependent on NF-κB Signaling Pathway?. Animals.

[33] Umezawa K. (2011). Possible role of peritoneal NF-kB in peripheral inflammation and cancer: Lessons from the inhibitor DHMEQ. Biomed Pharm..

[34] May M.J., Ghosh S. (1998). Signal transduction through NF-κB. Trends Immunol..

[35] Lind D.S., Hochwald S.N., Malaty J., Rekkas S., Hebig P., Mishra G., Moldawer L.L., Copeland III E.M., MacKay S. (2001). Nuclear factor-κB is upregulated in colorectal cancer. Surgery.

[36] Tripathi P., Aggarwal A. (2001). NF-kB transcription factor: A key player in the generation of immune response. Curr. Sci..

[37] Ahn B.N., Song M.H., Kim J.H., Kim K.H., Park K.K., Choi Y.S. (2012). Intra-peritoneal NF-kappaB decoy oligodeoxynucleotide decreases postoperative intra-abdominal adhesion. Korean J. Obstet. Gynecol..

[38] Sosinska P., Baum E., Mackowiak B., Staniszewski R., Jasinski T., Umezawa K., Breborowicz A. (2016). Inhibition of NF-kappaB with Dehydroxymethylepoxyquinomicin modifies the function of human peritoneal mesothelial cells. Am. J. Transl. Res..

[39] Alekseevna R.V., Pavlovich D.A., Evgenievich B.Y., Viktorovich N.S. (2017). Nuclear factor kappa B as a potential target for pharmacological correction endothelium-associated pathology. Res. Res. Pharm..

[40] Arjmand M.H. (2020). The association between visceral adiposity with systemic inflammation, oxidative stress, and risk of post-surgical adhesion. Arch. Physiol. Biochem..

[41] Dejban P., Nikravangolsefid N., Chamanara M., Dehpour A., Rashidian A. (2021). The role of medicinal products in the treatment of inflammatory bowel diseases (IBD) through inhibition of TLR4/NF-kappaB pathway. Phytother. Res..

[42] De Sousa Abreu R., Penalva L.O., Marcotte E., Vogel C. (2009). Global signatures of protein and mRNA expression levels. Mol. Biosyst..

[43] Vogel C., Marcotte E.M. (2012). Insights into the regulation of protein abundance from proteomic and transcriptomic analyses. Nat. Rev. Genet..

[44] Schoon H.-A., Schoon D., Klug E. (1992). Uterusbiopsien als Hilfsmittel für Diagnose und Prognose von Fertilitätsstörungen der Stute. Pferdeheilkunde.

[45] Da Costa R.R.P., Serrao P.M., Monteiro S., Pessa P., Robalo Silva J., Ferreira-Dias G. (2007). Caspase-3 mediated apoptosis and cell proliferation in the equine endometrium during the oestrous cycle. Reprod. Fertil. Dev..

[46] Gebhardt S., Merkl M., Herbach N., Wanke R., Handler J., Bauersachs S. (2012). Exploration of global gene expression changes during theestrous cycle in equine endometrium. Biol. Reprod..

[47] McDowell K.J., Adams M.H., Adam C.Y., Simpson K.S. (1999). Changes in equine endometrial oestrogen receptor and progesterone receptor mRNAs during the oestrus cycle, early pregnancy and after treatment with exogenous steroids. J. Reprod. Fertil..

[48] Honnens A., Weisser S., Welter H., Einspanier R., Bollwein H. (2011). Relationships between uterine blood flow, peripheral sex steroids, expression of endometrial estrogen receptors and nitric oxide synthases during the estrus cycle in mares. J. Reprod. Develop..

[49] Paech K., Webb P., Nisson S., Gustafsson J.-Å., Kushner P.J., Scanlan S. (1997). Differential Ligand Activation of Estrogen Receptors ERα and ERβ at AP1 Sites. Science.

[50] Hall J.M., McDonnell D.P. (1999). The Estrogen Receptor β-Isoform (ERβ) of the Human Estrogen Receptor Modulates ERα Transcriptional Activity and Is a Key Regulator of the Cellular Response to Estrogens and Antiestrogens. Endocrinology.

[51] Pettersson K., Grandien K., Kuiper G.G.J.M., Gustafsson J.-Å. (1997). Mouse Estrogen Receptor β Forms Estrogen Response Element-Binding Heterodimers with Estrogen Receptor α. Mol. Endocrinol..

[52] Cowley S.M., Hoare S., Mosselman S., Parker M.G. (1997). Estrogen Receptors α and β Form Heterodimers on DNA. J. Biol. Chem..

[53] Lubahn D.B., Moyer J.S., Golding T.S., Couse J.F., Korach K.S., Smithies O. (1993). Alteration of reproduction function but not prenatal sexual development after insertional disruption of the mouse estrogen receptor gene. Proc. Natl. Acad. Sci. USA.

[54] Pierro E., Minici F., Alesiani O., Miceli F., Proto C., Screpanti I., Lanzone A. (2001). Stromal-epithelial interactions modulate estrogen responsiveness in normal human endometrium. Biol. Reprod..

[55] Arnold J.T., Kaufman D.G., Seppala M., Lessey B.A. (2001). Endometrial stromal cells regulate epithelial cell growth in vitro: A new co-culture model. Hum. Reprod..

[56] Klymkowsky M.W., Bachant J.B., Domingo A. (1989). Functions of intermediate filaments. Cell Motil. Cytoskeleton.

[57] Rebordão M.R., Amaral A., Lukasik K., Szóstek-Mioduchowska A., Pinto-Bravo P., Galvão A., Skarzynski D.J., Ferreira-Dias G. (2018). Constituents of neutrophil extracellular traps induce in vitro collagen formation in mare endometrium. Theriogenology.

[58] Brasier A.R. (2010). The nuclear factor-B–interleukin-6 signalling pathway mediating vascular inflammation. Cardiovasc. Res..

[59] Ohkawa T., Ueki N., Taguchi T., Shindo Y., Adachi M., Amuroa Y., Hada T., Higashino K. (1999). Stimulation of hyaluronan synthesis by tumor necrosis factor- is mediated by the p50/p65 NF–B complex in MRC-5 myofibroblasts. Biochim. Biophys. Acta Mol. Cell Res..

[60] Caon I., Bartolini B., Moretto P., Parnigoni A., Carava E., Vitale D.L., Alaniz L., Viola M., Karousou E., De Luca G. (2017). Sirtuin 1 reduces hyaluronan synthase 2 expression by inhibiting nuclear translocation of NF-B and expression of the long-noncoding RNA HAS2–AS1. J. Biol. Chem..

[61] Tong W., Geng Y., Huang Y., Shi Y., Xiang S., Zhang N., Qin L., Shi Q., Chen Q., Dai K. (2015). In vivo identification and induction of articular cartilage stem cells by inhibiting NF-B signaling in osteoarthritis. Stem Cells.

[62] Chung S., Son M., Kim M., Koh E.S., Shin S.J., Park C.W., Kim S., Kim H.S. (2019). Inhibition of p300/CBP-associated factor attenuates renal tubulointerstitial fibrosis through modulation of NF-kB and Nrf2. Int. J. Mol. Sci..

[63] Schoon H.A., Schoon D., Klug E. (1997). Die Endometriumbiopsie bei der Stute im klinisch-gynäkologischen Kontext. Pferdeheilkunde.

